# Association of dietary inflammatory indices with sarcopenia and all-cause mortality in COPD patients

**DOI:** 10.3389/fnut.2024.1395170

**Published:** 2024-05-23

**Authors:** Qi Jiang, Zheru Ma, Jing Sun, Yang Li

**Affiliations:** ^1^Department of Respiratory Medicine, The First Hospital of Jilin University, Jilin, China; ^2^Department of Bone and Joint Surgery, Orthopedic Center, The First Hospital of Jilin University, Jilin, China; ^3^Department of Otolaryngology Head and Neck Surgery, The First Hospital of Jilin University, Jilin, China

**Keywords:** COPD, sarcopenia, DII, ASMI, NHANES

## Abstract

**Background:**

Sarcopenia frequently occurs as a comorbidity in individuals with COPD. However, research on the impact of Appendicular Skeletal Muscle Mass (ASM) on survival in COPD patients is scarce. Moreover, there is a lack of research on the association between dietary pro-inflammatory capacity and sarcopenia in COPD.

**Methods:**

We analyzed data from the National Health and Nutrition Examination Survey (NHANES) covering the years 1999 to 2006 and 2011 to 2018. We aimed to investigate the relationship between the Dietary Inflammatory Index (DII) and sarcopenia prevalence among adults diagnosed with COPD in the United States. Furthermore, we sought to explore the relationship between sarcopenia, ASMI, and all-cause mortality. The study included a total of 1,429 eligible adult participants, divided into four groups based on quartiles of DII, with adjustments for sample weights. Methodologically, we used multivariable logistic regression analyses and to examine the association between DII and sarcopenia. Additionally, we used restricted cubic spline (RCS) tests to evaluate potential non-linear relationships. To assess the effect of sarcopenia on overall all-cause mortality, we used Kaplan–Meier models and Cox proportional hazards models. Moreover, we used RCS analyses to investigate potential non-linear relationships between ASMI and all-cause mortality. Subgroup analyses were conducted to confirm the reliability of our study findings.

**Results:**

In our COPD participant cohort, individuals with higher DII scores were more likely to be female, unmarried, have lower educational attainment, and show lower ASMI. Using multivariable logistic regression models, we found a positive association between the highest quartile of DII levels and sarcopenia incidence [Odds Ratio (OR) 2.37; 95% Confidence Interval (CI) 1.26–4.48; *p* = 0.01]. However, analysis of RCS curves did not show a non-linear relationship between DII and sarcopenia. Throughout the entire follow-up period, a total of 367 deaths occurred among all COPD patients. Kaplan–Meier survival curves showed a significantly higher all-cause mortality rate among individuals with concurrent sarcopenia (*p* < 0.0001). Cox proportional hazards model analysis showed a 44% higher risk of all-cause mortality among COPD patients with sarcopenia compared to those without sarcopenia [Hazard Ratio (HR): 1.44; 95% CI 1.05–1.99; *p* < 0.05]. Additionally, our final RCS analyses revealed a significant non-linear association between ASMI levels and all-cause mortality among COPD patients, with a turning point identified at 8.32 kg/m^2^. Participants with ASMI levels above this inflection point had a 42% lower risk of all-cause mortality compared to those with ASMI levels below it (HR 0.58; 95% CI 0.48–0.7).

**Conclusion:**

We observed a significant association between concurrent sarcopenia and an increased risk of all-cause mortality in COPD patients within the United States. Moreover, ASMI demonstrated a non-linear association with all-cause mortality, with a critical threshold identified at 8.32 kg/m^2^. Our findings also revealed an association between DII and the presence of sarcopenia. Consequently, further investigations are warranted to explore the feasibility of dietary DII adjustments as a means to mitigate muscle wasting and enhance the prognosis of COPD.

## Introduction

Chronic obstructive pulmonary disease (COPD) significantly contributes to the global disease burden, mortality rates, and healthcare resource utilization (1). Globally, it ranks as the fourth leading cause of death, affecting approximately 12% of the world’s population (2). COPD comprises a range of chronic inflammatory respiratory conditions characterized by airflow obstruction and respiratory impairment. Primary causative factors often involve a history of smoking and exposure to environmental pollutants. Additionally, COPD often leads to various serious comorbidities, such as lung cancer, coronary heart disease, heart failure, renal dysfunction, hyperuricemia, and muscle wasting (3). These interrelated comorbidities, along with a diminished quality of life, impose additional burdens on patients, increasing the risk and mortality associated with these concurrent conditions (4).

Sarcopenia, known as muscle wasting, is a progressive systemic skeletal muscle disease that raises the risk of falls, fractures, and mortality rates, significantly contributing to physical disability and frailty (5). In 2010, the European Working Group on Sarcopenia in Older People (EWGSOP) established a universally acknowledged definition of sarcopenia (6). In early 2018, the group reconvened (EWGSOP2), providing updated guidelines and recommendations on sarcopenia definition, diagnosis, measurement, and associated risks, informed by scientific and clinical evidence from the past decade (7). The latest recommendations from EWGSOP2 aim to raise awareness of sarcopenia and its risks, advocating for early detection and treatment and encouraging additional research to prevent or delay adverse health outcomes for patients and healthcare systems. While sarcopenia has traditionally been linked with aging, it is now recognized to initiate early in life (8). Literature evidence associating birth weight, infant growth, and adult grip strength underscores the lasting impact of prenatal and postnatal muscle development, influencing muscle function via fiber size, quantity, and satellite cell activity (9). Currently, sarcopenia etiology is classified into primary (aging-related) and secondary (disease-related, lack of activity, and malnutrition). Sarcopenia can arise secondary to systemic diseases, notably those provoking inflammatory processes like malignancies, COPD, and cardiovascular diseases, among others. Moreover, inadequate physical activity and insufficient energy or protein intake can contribute to sarcopenia development (7). Due to the multifactorial nature of sarcopenia development, interactions among these factors can exacerbate its progression. As sarcopenia can be secondary to COPD and worsen COPD symptoms by affecting skeletal, smooth, and cardiac muscle function, diaphragmatic contraction, and patient activity levels, it can lead to respiratory failure and increase mortality among COPD patients (10). The interaction between COPD and sarcopenia creates a vicious cycle, where sarcopenia originating from COPD exacerbates COPD symptoms and accelerates disease progression. Considering this complex relationship, it is crucial to investigate the high-risk factors that predispose COPD patients to sarcopenia. This exploration offers the potential to reveal new insights into managing and treating COPD patients, improving their condition.

Targeted nutritional interventions, like high-protein diets, show promise in improving respiratory muscle strength, physical performance, overall health status, and quality of life in elderly COPD patients (11). These interventions can aid in the rehabilitation process (12). Conversely, age-related chronic low-grade systemic inflammation significantly contributes to sarcopenia onset, with dietary factors playing a crucial role (13). Certain nutrients, foods, and dietary patterns are associated with inflammation biomarkers. Yet, the complex interaction and mechanisms between inflammation and diet require further exploration (14). Considering these factors, the Dietary Inflammatory Index (DII) emerges as a valuable tool for assessing the inflammatory potential of dietary components (15, 16). The DII assesses the impact of nutrients and foods on six key inflammatory markers (IL-1β, IL-4, IL-6, IL-10, TNF-α, and CRP), computing pro-inflammatory and anti-inflammatory scores for each dietary parameter to generate an overall inflammation effect score (17). In recent years, the DII has gained considerable attention in clinical research, enabling investigations into the relationship between dietary inflammatory potential and various diseases (15). In a notable study by Gojanovic et al. (18), adherence to a pro-inflammatory diet was associated with reduced muscle mass and poorer muscle function. These dietary patterns may undermine muscle health and function, increasing the risk of sarcopenia. Remarkably, there is a lack of studies exploring the potential relationship between the dietary inflammatory index and sarcopenia in individuals with COPD. Therefore, exploring the association between the dietary inflammatory index and both all-cause mortality and sarcopenia-related mortality in COPD patients is clinically significant.

The study utilized data from the National Health and Nutrition Examination Survey (NHANES), covering 1999 to 2006 and 2011 to 2018. The primary objective is to investigate the association between DII and sarcopenia, as well as the link between sarcopenia, muscle mass, and all-cause mortality in individuals with COPD. The overarching goal of this research is to provide new insights and empirical evidence to enhance survival outcomes in COPD patients by implementing dietary management strategies. This study aims to pave the way for more effective nutritional interventions tailored to the COPD population in the future.

## Materials and methods

### Study design and data source

The study utilizes NHANES data from 1999 to 2006 and 2011 to 2018. NHANES is administered by the National Center for Health Statistics (NCHS) under the guidance of the Centers for Disease Control and Prevention (CDC). Trained professionals conduct household interviews or administer examinations in mobile examination centers for data collection in NHANES. The survey includes cross-sectional interviews, physical examinations, and laboratory analyses collected from a complex, multi-stage, stratified, clustered probability sample representing the US population (19). The survey protocol is ethically overseen by the Institutional Review Board of the CDC, and informed consent is secured from all participants. The NHANES data are free and available on the Web (20). During NHANES cycles from 1999 to 2006 and 2011 to 2018, individuals aged 20 and above participated in research conducted by NHANES. NHANES methodology employs dual-energy X-ray absorptiometry (DXA) to measure body composition due to its speed, cost-effectiveness, and minimal radiation exposure (20). Individuals weighing over 136 kg or measuring over 192 cm were excluded due to DXA scanning limitations. Pregnant women and individuals with recent use of radiographic contrast agents or radiotherapy within the past 7 days were also excluded from the analysis.

### Body composition, DXA, and definition of low muscle mass

NHANES surveys from 1999 to 2006 and 2011 to 2018 captured body composition parameters including height (cm), weight (kg), and waist circumference (cm). BMI was calculated using height and weight measurements. DXA scans were conducted on individuals aged 8 to 59 during NHANES 2011–2018 using the Hologic Discovery A densitometer. Within DXA scans, non-fat and non-bone mass components were classified as skeletal muscle, and Appendicular Skeletal Muscle Mass (ASM) represented the sum of lean soft tissue in the four limbs. According to EWGSOP2 recommendations (7), key assessments for identifying sarcopenia in clinical practice and research encompass: (1) Questionnaire screening (21). (2) Muscle strength evaluation, including grip strength measurement (22), chair stand test, etc. (3) Quantitative assessment of muscle mass, utilizing techniques like DXA for ASM measurement (23–25) and bioelectrical impedance analysis (BIA) for predicting total body skeletal muscle mass (SMM) or ASM (26–29). (4) Physical performance assessment, evaluating gait speed and conducting a 400-meter walk test to gauge walking ability and endurance (22), among others. Since muscle mass is correlated with body size, with larger individuals typically having greater muscle mass, quantifying muscle mass can be adjusted using various methods to normalize SMM or ASM absolute levels, such as height squared (ASM/height^2^), body weight (ASM/body weight), or body mass index (ASM/BMI) (30). Despite ongoing controversy surrounding the adjustment of ASM for body size across diverse populations, we opted to use ALM/BMI for diagnosing sarcopenia. ALM/BMI defines low muscle mass with gender-specific thresholds: According to data from The Foundation for the National Institutes of Health(FNIH) Biomarkers Consortium Sarcopenia Project, low muscle mass is diagnosed if ALM/BMI is <0.789 in males, and < 0.512 in females (31). We then evaluated muscle mass using ASM/height^2^ [Appendicular Skeletal Muscle Index, ASMI (32)] as a continuous variable for further analysis.

### Definition of COPD

COPD diagnosis relies on participant self-reporting confirmed by a physician. COPD diagnosis is confirmed if participants answer positively to questions regarding physician-diagnosed COPD, related conditions like emphysema or chronic bronchitis. Participants answering “yes” are classified as COPD-positive, while those answering “no” are classified as COPD-negative. Those with an FEV1/FVC ratio less than 0.7 on pulmonary function testing are also classified as COPD-positive. The COPD group also encompasses individuals aged 40 and above with a history of smoking or chronic bronchitis who are using specific medications, such as selective phosphodiesterase-4 inhibitors, mast cell stabilizers, leukotriene modifiers, and inhaled corticosteroids.

### DII definition

The method for calculating the DII was introduced by Shivappa et al. (15). In this study, DII served as the exposure variable, and dietary information was obtained via a 24-h dietary recall conducted on the initial day. DII calculation incorporated 26 dietary parameters, encompassing carbohydrates, protein, total fat, saturated fat, polyunsaturated fatty acids (PUFA), n-3 fatty acids, cholesterol, energy, alcohol, fiber, folate, iron, magnesium, zinc, selenium, monounsaturated fatty acids (MUFA), caffeine, niacin, riboflavin, thiamin, β-carotene, vitamins A/B6/B12/C/E (33). Initially, the mean intake of each nutrient among participants was calculated, then subtracted from the global mean intake, and divided by the standard deviation to compute z-scores. Next, z-scores underwent transformation into percentiles, doubled, and subsequently subtracted by “1” to center the data. Centered percentile values for each dietary parameter were multiplied by their respective inflammatory effect scores to yield “specific DII scores” for each food parameter. Lastly, these values were summed to determine individual “overall DII scores” (34).

### Covariates

In this study, we used data collected from NHANES. Data in NHANES is collected through standardized questionnaire methods (20), such as gender, age, race, education level, poverty index, marital status, diabetes, cardiovascular disease, and hypertension history, through household interviews. Physical data, such as weight, height, and blood pressure, were obtained during examinations at mobile examination centers. BMI was computed by dividing weight in kilograms by height in meters squared. Race categories included Mexican American, non-Hispanic white, non-Hispanic black, and other races. Education level was segmented into three groups: individuals without high school completion, those with high school completion or equivalent, and those with higher education. Family income was classified into low income, middle income, and high income brackets (PIR < 1.30, 1.30–3.49, ≥3.50). Marital status included categories of married/living with partner, never married, and widowed/divorced/separated. Diabetes diagnosis relied on self-reported history or medication use. Hypertension was diagnosed if systolic blood pressure was over 140 mmHg, diastolic blood pressure exceeded 90 mmHg, individuals were taking antihypertensive medications, or there was a self-reported history of hypertension. Cardiovascular disease (CVD) history was established by physician diagnosis of myocardial infarction, angina pectoris, coronary heart disease, or stroke.

### Statistical methods

Analyses were performed using R software (version 4.1.1). Due to the NHANES survey’s complex design, we adhered to NHANES recommendations and applied eight-year cycle weights, stratification, and clustering to our analyses. Categorical variables were expressed as numbers and weighted percentages, while continuous variables were presented as weighted means (standard errors). Participants were grouped into four quartiles based on DII scores. Weighted linear regression and design-adjusted chi-square tests were used to compare baseline characteristics between groups for continuous and categorical variables, respectively. Multivariable weighted regression analyses were conducted to evaluate the independent association between DII and sarcopenia, adjusting for potential confounders and obtaining odds ratios (OR) and 95% confidence intervals (CI). Model 1 was unadjusted; Model 2 adjusted for age, gender, race, education, poverty index, marital status; Model 3 further adjusted for history of diabetes, cardiovascular disease, and hypertension based on Model 2. Additionally, to explore the potential non-linear relationship between DII and sarcopenia, we used restricted cubic spline (RCS) models, adjusting for potential confounders. Kaplan–Meier curves and Cox regression models were used to assess the relationship between sarcopenia and all-cause mortality in COPD patients. RCS was also used to examine the non-linear relationship between ASMI and all-cause mortality. If the relationship was non-linear, a recursive algorithm was used to calculate ASMI and all-cause mortality separately. Two-piece Cox proportional hazards models were then applied to investigate the association between ASMI and all-cause mortality risk on both sides of the inflection point. Finally, subgroup analyses were performed to validate the above results. Subgroup analyses were based on the following considerations: (1) Previous studies have indicated differences between subgroups, including gender, age, and race, as demographic factors and lifestyle may influence muscle mass. (2) Various biological mechanisms, especially in chronic diseases like diabetes, hypertension and CVD, may play significant roles. Therefore, subgroup analyses were conducted among individuals with and without these conditions. A *p*-value <0.05 indicated statistical significance.

## Results

### Baseline characteristics of COPD participants

Among the initial 80,630 participants, individuals without a diagnosis of COPD were excluded (*n* = 77,309), along with those with missing muscle mass index (*n* = 1,846) and DII data (*n* = 46). A total of 1,429 participants met the inclusion criteria, and Table 1 presents the characteristics of these participants. Participants with higher dietary validation index scores comprised more females, unmarried individuals, individuals with lower education levels, sarcopenic individuals, and those with lower ASMI. In order to ensure the study’s stability and eliminate the influence of confounding factors, we conducted additional subgroup analyses of ASMI using DII as both a continuous variable and a four-category grouped variable. The results indicated no interaction effect of DII on ASMI across all subgroups (Supplementary Table S1).

**Table 1:** Baseline characteristics of participants with COPD according to DII.

Variable	Total (*N* = 1,429)	Q1 (*N* = 359) [−4.53,0.76]	Q2 (*N* = 355) [0.76, 2.27]	Q3 (*N* = 357) [2.27,3.19]	Q4 (*N* = 358) [3.19, 5.19]	*p*-value
Age, years, *n* (%)						0.24
≥60	388 (18.68)	108 (19.86)	97 (18.39)	92 (18.92)	91 (17.25)	
≥40and<60	668 (51.32)	150 (45.84)	171 (55.49)	167 (48.48)	180 (56.94)	
<40	373 (29.99)	101 (34.30)	87 (26.11)	98 (32.60)	87 (25.81)	
Gender, *n* (%)						< 0.0001
Male	543 (35.69)	180 (47.03)	145 (40.71)	117 (31.08)	101 (21.51)	
Female	886 (64.31)	179 (52.97)	210 (59.29)	240 (68.92)	257 (78.49)	
Race, *n* (%)						0.95
White	867 (76.58)	219 (77.25)	213 (75.83)	229 (77.76)	206 (75.16)	
Black	249 (8.87)	60 (8.18)	67 (9.85)	50 (7.70)	72 (10.04)	
Mexican American	144 (4.18)	45 (5.29)	30 (3.73)	31 (3.83)	38 (3.72)	
Others	169 (10.37)	35 (9.28)	45 (10.59)	47 (10.70)	42 (11.08)	
Marriage, *n* (%)						0.02
Married/Living with partner	763 (55.87)	208 (61.11)	181 (54.86)	193 (56.26)	181 (55.05)	
Widowed/divorced/separated	429 (27.15)	87 (20.67)	117 (31.58)	100 (25.63)	125 (34.46)	
Never married	215 (15.06)	55 (18.22)	50 (13.56)	62 (18.12)	48 (10.49)	
Poverty, *n* (%)						< 0.0001
High (>3.5)	302 (27.24)	98 (36.23)	86 (32.37)	67 (26.34)	51 (18.00)	
Middle (>1.3and ≤ 3.5)	501 (36.09)	140 (44.25)	122 (36.16)	128 (36.43)	111 (34.13)	
Low (≤1.3)	534 (31.64)	98 (19.52)	120 (31.48)	141 (37.23)	175 (47.87)	
Education, *n* (%)						0.01
>High school	656 (49.69)	188 (57.21)	159 (49.94)	156 (44.76)	153 (46.19)	
High school	350 (27.42)	90 (28.38)	81 (24.25)	98 (32.59)	81 (23.64)	
< High school	422 (22.79)	81 (14.42)	114 (25.81)	103 (22.65)	124 (30.17)	
Diabetes, *n* (%)						0.89
Yes	212 (12.00)	54 (11.58)	44 (11.07)	52 (12.12)	62 (13.50)	
No	1,216 (87.87)	304 (88.42)	311 (88.93)	305 (87.88)	296 (86.50)	
Hypertension, *n* (%)						0.11
Yes	728 (45.29)	173 (40.66)	195 (49.70)	185 (49.55)	175 (41.02)	
No	701 (54.71)	186 (59.34)	160 (50.30)	172 (50.45)	183 (58.98)	
CVD, *n* (%)						0.18
Yes	288 (15.39)	63 (12.25)	66 (14.25)	71 (17.60)	88 (17.90)	
No	1,141 (84.61)	296 (87.75)	289 (85.75)	286 (82.40)	270 (82.10)	
Sarcopenia, *n* (%)						0.02
Yes	246 (12.72)	47 (8.17)	56 (10.86)	66 (16.15)	77 (16.32)	
No	1,183 (87.28)	312 (91.83)	299 (89.14)	291 (83.85)	281 (83.68)	
ASMI, Kg/m^2^, Mean (SD)	7.40 (0.06)	7.68 (0.11)	7.28 (0.10)	7.40 (0.11)	7.17 (0.14)	0.01

### DII and sarcopenia in COPD

**Figure 1:**
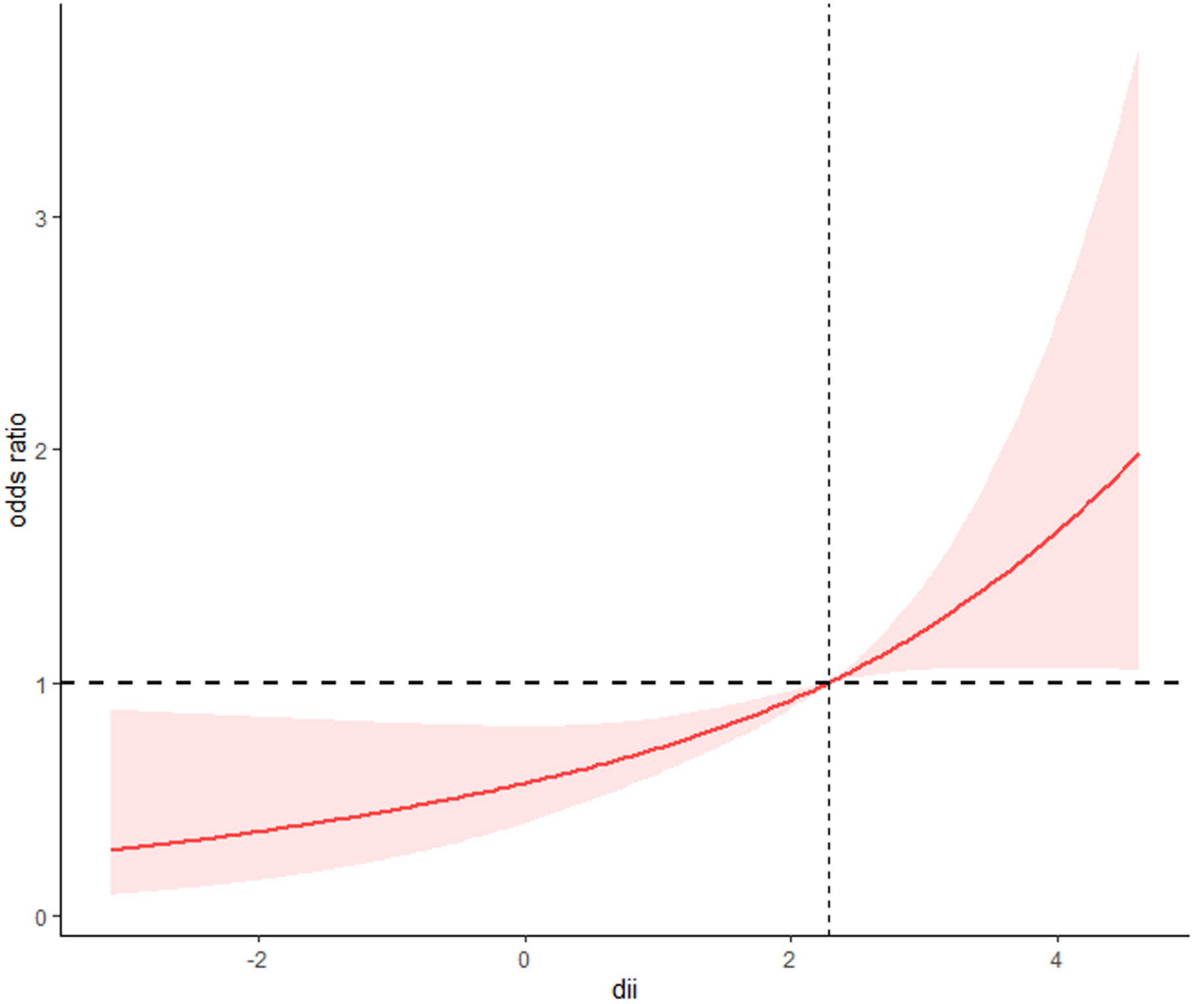
The restricted cubic spline model showed a dose–response relationship between dietary DII intake and sarcopenia. P for non-linearity = 0.98.

Three logistic regression models were developed, taking into account weighting considerations. Model 1 performed univariate analysis of DII and sarcopenia among COPD patients. Model 2 included variables such as age, gender, race, poverty index, education level, and marital status. Model 3 additionally incorporated histories of hypertension, diabetes, and cardiovascular disease, building on the variables in Model 2. All three models revealed a positive correlation between DII scores and sarcopenia prevalence in COPD patients. Specifically, in fully adjusted Model 3, logistic regression analysis showed that compared to DII quartile 1 (Q1), the multivariate adjusted OR and 95% CI for Q3 and Q4 were 2.32 (1.30, 4.15) and 2.37 (1.26, 4.48), respectively (Table 2). A clear dose–response relationship between DII scores and sarcopenia in COPD patients was identified (Figure 1). This analysis demonstrated a linear association between these variables, with a non-linear *p*-value of 0.98, indicating that as DII scores in dietary intake increase, sarcopenia prevalence rises accordingly.

**Table 2:** OR and 95% CI for sarcopenia according quartile of DII intake.

DII	OR (95%CI), *p*-value					
	Model 1		Model 2		Model 3	
Character	95%CI	*p*	95%CI	*p*	95%CI	*p*
Q1 [−4.53, 0.76]	ref		ref		ref	
Q2 [0.76, 2.27]	1.37(0.71,2.63)	0.34	1.39(0.75,2.58)	0.29	1.43(0.75,2.73)	0.27
Q3 [2.27, 3.19]	2.17(1.32,3.56)	**0.003**	2.48(1.40,4.39)	**0.002**	2.32(1.30,4.15)	**0.005**
Q4 [3.19, 5.19]	2.19(1.24,3.88)	**0.01**	2.28(1.22,4.26)	**0.01**	2.37(1.26,4.48)	**0.01**
p for trend		**0.002**		**0.003**		**0.005**

OR, odds ratio; CI, confidence interval; Q, quartile. Model 1: no adjustment. Model 2: adjusted for age, gender, race, poverty, education, marriage. Model 3: adjusted for age, gender, race, poverty, education, marriage, Diabetes, CVD, hypertension. The lowest quartile of dietary DII intake was used as the reference group. Bold values indicate statistically significant *p*-values.

### Sarcopenia/ASMI and all-cause mortality in COPD patients

**Figure 2:**
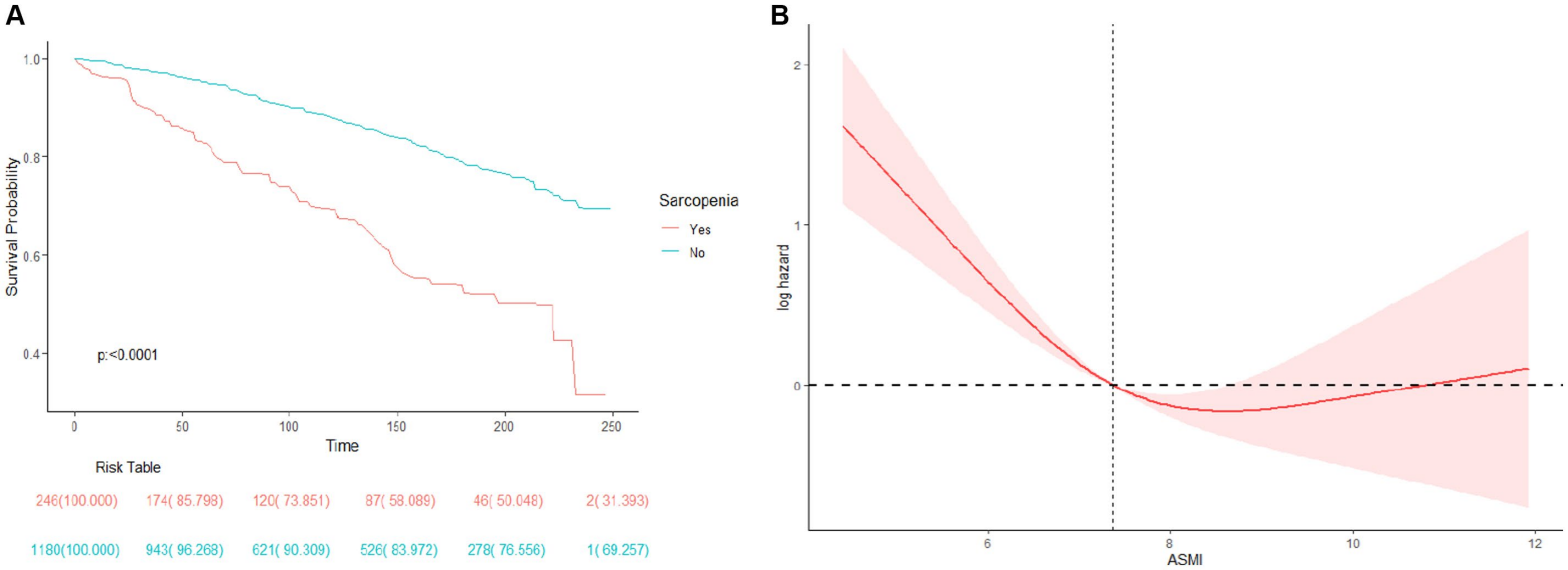
**(A)** Survival (Kaplan–Meier) curves for All-cause mortality according to history of Sarcopenia in COPD patients. **(B)** Association between ASMI levels and all-cause mortality in patients with COPD.

A total of 367 deaths were recorded over the entire follow-up period, with a median follow-up time of 106 months. Kaplan–Meier curves showed a significantly higher all-cause mortality rate in participants with sarcopenia (*p* < 0.0001) (Figure 2A). Further adjusting for covariates, the Cox proportional hazards model showed that COPD patients with sarcopenia had a 44% higher risk of all-cause mortality compared to those without sarcopenia (HR 1.44; 95%CI 1.05–1.99; *p* = 0.03) (Table 3). RCS results also showed a non-linear relationship between ASMI levels and all-cause mortality in COPD patients (p non-linear <0.001) (Figure 2B). Threshold effect analysis suggested an ASMI threshold of 8.32 (Table 4). Below the ASMI threshold of 8.32, each unit increase was associated with a 42% reduction in all-cause mortality risk (HR 0.58; 95%CI 0.48–0.7; *p* < 0.0001), whereas no such association was observed above this threshold (HR 0.9; 95%CI 0.61–1.34; *p* = 0.61).

**Table 3:** HR (95% CI) for all-cause mortality according to COPD patients with concomitant sarcopenia.

Sarcopenia	HR(95%CI), *p*-value					
	Model 1		Model 2		Model 3	
Character	95%CI	*p*	95%CI	*p*	95%CI	*p*
No	ref		ref		ref	
Yes	3.18(2.36,4.28)	<0.0001	1.62(1.18, 2.23)	0.003	1.44(1.05, 1.99)	0.03
p for trend		<0.0001		0.003		0.03

Model 1: no adjustment. Model 2: adjusted for age, gender, race, poverty, education, marriage. Model 3: adjusted for age, gender, race, poverty, education, marriage, Diabetes, CVD, hypertension.

**Table 4:** Effect of ASMI level on survival: adjusted hazard ratios from segmented Cox (adjusted for age, gender, race, poverty, education, marriage, diabetes, CVD, hypertension).

Characteristic	Adjusted HR	95% CI1	*p*-value
ASMI≥8.32	0.9	(0.61, 1.34)	0.61
ASMI<8.32	0.58	(0.48,0.7)	<0.0001

### Stratified analysis

**Figure 3:**
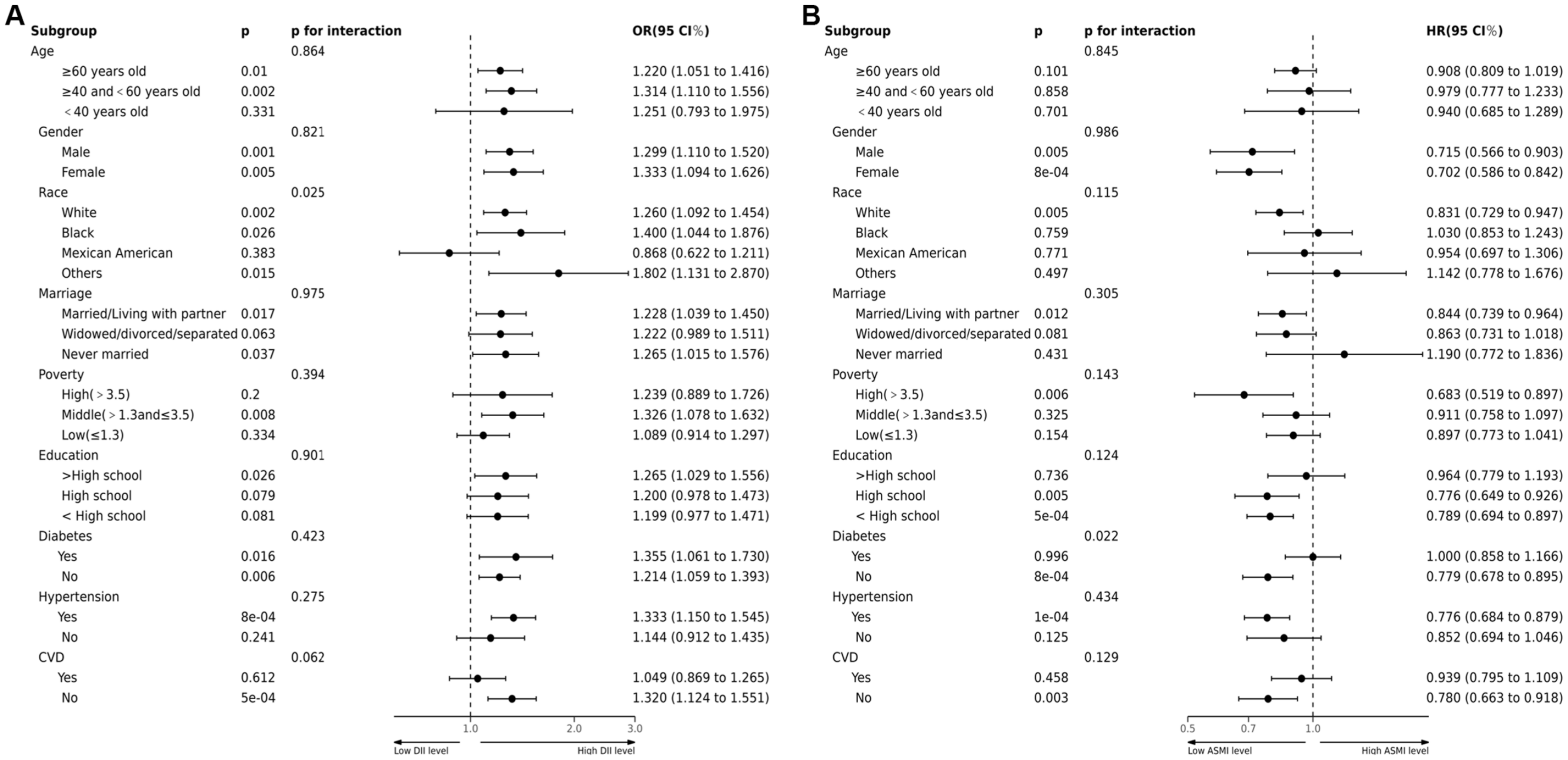
**(A)** Forest plots of stratified analyses of DII and sarcopenia in COPD. Age, gender, race, marriage, poverty, education and history of hypertension, CVD or diabetes were all adjusted except the variable itself. **(B)** Forest plots of stratified analyses of sarcopenia and all-cause mortality. Age, gender, race, marriage, poverty, education and history of hypertension, CVD or diabetes were all adjusted except the variable itself.

This study conducted a stratified analysis to investigate the impact of demographic characteristics and comorbidities on the relationship between DII and sarcopenia incidence, as well as ASMI and all-cause mortality rate among COPD patients. Sarcopenia incidence was similar across various subgroups stratified by age, gender, marital status, education, poverty, hypertension history, and diabetes history. No significant interaction was found between the DII index and stratification variables. However, interactions were observed between DII and race (Figure 3A). In terms of the relationship between ASMI and all-cause mortality, an interaction was observed between ASMI and history of diabetes, with no significant interactions found with other stratification variables (Figure 3B).

## Discussion

Muscle loss has become a significant concern for quality of life in patients with various chronic diseases (35). While the association between COPD and sarcopenia has gained research attention in recent years (12, 36–38), to our knowledge, this study is the first to explore the link between DII and sarcopenia in COPD patients. It reveals that COPD patients with a pro-inflammatory diet are at higher risk of sarcopenia. Our study revealed a dose–response relationship between DII and sarcopenia in COPD patients, suggesting that higher DII index in dietary intake among COPD patients is associated with increased incidence of sarcopenia. Chen et al. (39) first reported the correlation between dietary inflammation index scores and muscle mass in older adults, confirming that an elevated DII index in the diet is linked to reduced muscle mass in the general population. Comparison with their data showed that in the highest quartile of DII index, COPD patients had significantly lower ASMI (7.17 ± 0.14) compared to the general population (7.40 ± 1.31), suggesting that a higher prevalence of pro-inflammatory diets among COPD patients may increase the risk of sarcopenia. Additional research on the association between sarcopenia and overall mortality in COPD patients showed that in the COPD population, for every unit increase when ASMI is below 8.32, the overall mortality risk decreases by 42%. This emphasizes the strong correlation between sarcopenia and mortality rates in COPD patients. Sepúlveda-Loyola et al.’s (40) meta-analysis results show that sarcopenia negatively affects various COPD-related clinical outcomes, underscoring the importance of studying COPD and sarcopenia. Our study findings suggest that modifying the pro-inflammatory diet in COPD patients can partially mitigate the decline in muscle mass, albeit with limitations. Further investigation into the factors influencing sarcopenia onset in COPD patients is needed for interventions aimed at enhancing patient quality of life.

COPD, as an inflammatory disease, has long been a focus of interest for exploring anti-inflammatory therapy (41). While current research has not conclusively shown the effective improvement of the disease process with anti-inflammatory drugs, inflammation remains a central focus of COPD pathogenesis research, and anti-inflammatory treatment retains significant research value (42, 43). Currently, COPD treatment primarily involves pharmacotherapy and non-pharmacological interventions. Commonly used drugs include β2 agonists, anticholinergic drugs, corticosteroids, and antibiotic prophylaxis (44). Non-pharmacological interventions mainly involve smoking cessation, long-term oxygen therapy, pulmonary rehabilitation, and nutritional support. However, current treatments can only alleviate symptoms and delay disease progression without curing COPD. Consequently, research focus has shifted to studying COPD-related extrapulmonary complications, including coronary heart disease, heart failure, renal insufficiency, and muscle wasting. As research progresses, extrapulmonary manifestations are increasingly recognized as significant contributors to functional decline in COPD patients (45). Functional impairment in COPD patients is linked to muscle weakness and weight loss (46, 47). Several assessment methods have shown reduced muscle mass in COPD patients (48). Furthermore, according to relevant literature, patients with moderate to severe COPD or those experiencing acute exacerbations are more prone to muscle mass and strength loss, particularly in the lower limbs (49). Severe sarcopenia can worsen COPD symptoms and increase the risk of death in patients.

Sarcopenia, also referred to as “muscle depletion,” is characterized by the progressive decline in muscle strength, mass, and function, resulting in reduced physical capacity and heightened vulnerability to disability, falls, and mortality, frequently worsened by concurrent conditions like cardiovascular disease, COPD, and chronic kidney disease (50). Sarcopenia’s underlying pathophysiological mechanisms are intricate, involving multifaceted interactions among various physiological systems (51), The principal mechanisms include: (1) Structural changes in skeletal muscle, characterized by disruptions in muscle homeostasis and neuronal degeneration leading to satellite cell aging, loss of type II fibers (hypoplasia), and diminished functional motor units (52, 53); (2) Muscle atrophy, attributed to metabolic disturbances such as skeletal muscle lipid or myocellular infiltration, alongside a pro-inflammatory state, insulin resistance, and glucose tolerance abnormalities (54); (3) Impedance of muscle homeostasis and synthesis metabolism, resulting from inhibition of muscle synthesis-related signaling pathways like serine/threonine kinase Akt/mTOR or disruptions in amino acid absorption, transport, and protein synthesis due to other metabolic diseases, leading to synthesis metabolic resistance, protein imbalance, and consequent muscle atrophy (55, 56); (4) Inflammation and mitochondrial dysfunction, marked by the chronic accumulation of pro-inflammatory and inflammatory factors such as CRP, IL-1, IL-6, and TNF-α, inducing skeletal muscle mitochondrial dysfunction, cellular degradation, heightened reactive oxygen species production, activation of the ubiquitin-proteasome cascade, and muscle protein hydrolysis, thereby causing muscle wasting. This mechanism is associated with an elevated risk of various chronic ailments, including heart failure, atherosclerotic heart disease, and COPD (57, 58); (5) Neurological pathways, where age-related degenerative alterations in the nervous system are implicated in muscle wasting development. The neuromuscular system’s integrity significantly influences muscle contraction strength and speed. Although the precise mechanisms behind neurological system-induced muscle wasting are not fully elucidated, muscle wasting due to muscle weakness from impaired neuromuscular system integrity is deemed a pivotal mechanism of muscle wasting (59). Among elderly COPD patients, sarcopenia correlates with hastened COPD advancement, heightened mortality risk, increased falls, and diminished quality of life. Despite the intricate pathophysiological mechanisms, sarcopenia’s fundamental etiology involves an imbalance between muscle synthesis and breakdown metabolism, sometimes accompanied by neuronal degeneration. Intrinsic molecular factors like aging, inflammatory response, chronic diseases, malnutrition, and reduced mobility contribute to sarcopenia development. Screening for sarcopenia, particularly in patients with chronic diseases like COPD, holds significant clinical relevance. Early identification of sarcopenia and timely intervention may mitigate the advancement of muscle disorders, thereby alleviating the adverse effects on COPD patient outcomes.

Inflammatory processes are a major contributor to accelerated muscle mass loss, particularly in COPD patients, where inflammation plays a crucial role in disease development and progression. Research by Buchmann et al. (57) has shown that elevated serum levels of inflammatory markers like IL-1β, IL-6, and CRP are linked to muscle weakness in critically ill patients, underscoring inflammation’s significant role in sarcopenia development. Conversely, diet serves as a vital systemic regulator of inflammation, directly impacting muscle loss. Research findings from a six-year multicenter cross-sectional study by Abete et al. (60) suggest that adhering to the Mediterranean diet, consuming specific nutrients (e.g., vitamin C), and engaging in physical activity act as protective factors against sarcopenia development. This highlights the potential strategy of combining a healthy diet with exercise to counter age-related sarcopenia. Recent academic research indicates that dietary fiber intake may have therapeutic effects on inflammation (61). The DII serves as a valuable tool for assessing the overall inflammatory potential of the diet by considering the inflammatory properties of various dietary components. The inclusion of DII in relevant studies is becoming more common, providing valuable insights for informing clinical practice. Given that inflammation plays a critical role in COPD development, and current theories propose that a high dietary fiber intake may attenuate age-related sarcopenia progression by reducing inflammation (62). However, prior to our study, research on the mediating role of DII in the relationship between COPD and sarcopenia risk among patients was lacking, and our study is the first to delve into this area.

This study has limitations. Firstly, cross-sectional studies allow us to investigate the association between DII and sarcopenia, not causal relationships. Further prospective studies or clinical trials are needed to confirm this. Secondly, sarcopenia involves a decrease in skeletal muscle mass accompanied by functional decline. In this study, we assessed sarcopenia solely through low muscle mass. Additionally, controversy persists regarding the adjustment of ASM for body size in different populations, and the diagnostic criteria for sarcopenia may vary, potentially affecting the diagnosis. Therefore, the diagnostic criteria for sarcopenia remain an area for future research. Moreover, due to the lack of relevant information in the NHANES dataset, muscle function was not assessed. Therefore, future research investigating the association between muscle function and DII is necessary. Thirdly, the DII score relies on food frequency questionnaires to obtain dietary intake data for calculation, which may be influenced by recall and misclassification biases. However, these biases may not differ between the COPD and non-COPD groups. Nonetheless, future studies may benefit from serum-based analyses to more accurately determine the direct relationship between DII levels and sarcopenia. Despite these limitations, the study has several strengths. Notably, it is the first to confirm the relationship between DII and sarcopenia in COPD patients based on the public NHANES database. The large and unstructured sample enhances the persuasiveness and applicability of our results. Additionally, we conducted multivariable regression and subgroup analyses to control for sociodemographic factors and some sarcopenia risk factors. Lastly, our study found an association between concomitant sarcopenia and higher all-cause mortality risk in COPD patients, suggesting the need for targeted improvements in muscle mass to enhance the quality of life and survival duration of these patients in the future.

## Conclusion

Our study revealed that in COPD patients, the presence of sarcopenia is linked to an increased risk of all-cause mortality. Furthermore, ASMI demonstrates a non-linear association with all-cause mortality, with a threshold of 8.32 kg/m^2^. Furthermore, a correlation exists between DII and the onset of sarcopenia in individuals with COPD. These results imply that lowering the DII score in the diet of COPD patients could offer potential benefits in preventing and managing the progression of COPD.

## Data availability statement

The original contributions presented in the study are included in the article/Supplementary material, further inquiries can be directed to the corresponding author.

## Author contributions

QJ: Conceptualization, Data curation, Software, Writing – original draft, Writing – review & editing. ZM: Conceptualization, Writing – original draft. JS: Writing – original draft. YL: Supervision, Writing – review & editing.
