# Supplementary material for: Association of dietary inflammatory indices with sarcopenia and all-cause mortality in COPD patients
Source: Front Nutr. 2024 May 23;11:1395170. doi: 10.3389/fnut.2024.1395170 (PMC11153795; doi:10.3389/fnut.2024.1395170)
Supplement: Supplementary file 1 [file Table_1.DOCX]

| Subgroup | DII as continuous variable | | | DII Stratified by quartile | | | | | | | |
| --- | --- | --- | --- | --- | --- | --- | --- | --- | --- | --- | --- |
|  | 95% CI | p | p for interaction | Q1 | Q2 | p | Q3 | p | Q4 | p | p for interaction |
| Age |  |  | 0.268 |  |  |  |  |  |  |  | 0.439 |
| ≥60 years old | -0.122(-0.244,0.000) | 0.051 |  | ref | -0.535(-1.104,0.034) | 0.065 | -0.328(-0.918,0.262) | 0.269 | -0.52(-1.112,0.072) | 0.084 |  |
| ≥40 and＜60 years old | -0.106(-0.231,0.019) | 0.095 |  | ref | -0.483(-0.980, 0.014) | 0.057 | -0.493(-1.001, 0.016) | 0.057 | -0.783(-1.253,-0.312) | 0.001 |  |
| ＜40 years old | 0.007(-0.114,0.128) | 0.905 |  | ref | -0.182(-0.734,0.370) | 0.513 | 0.051(-0.521,0.622) | 0.86 | -0.01(-0.668,0.648) | 0.977 |  |
| Gender |  |  |  |  |  |  |  |  |  |  | 0.176 |
| Male | 0.02(-0.058,0.099) | 0.604 | 0.815 | ref | -0.53(-0.849,-0.211) | 0.001 | 0.15(-0.239, 0.539) | 0.445 | -0.178(-0.647, 0.292) | 0.453 |  |
| Female | 0.006(-0.086,0.098) | 0.898 |  | ref | -0.13(-0.555,0.294) | 0.544 | -0.09(-0.474,0.293) | 0.641 | -0.072(-0.499,0.354) | 0.737 |  |
| Race |  |  |  |  |  |  |  |  |  |  | 0.447 |
| White | -0.097(-0.191,-0.003) | 0.042 | 0.367 | ref | -0.515(-0.856,-0.175) | 0.003 | -0.323(-0.713, 0.066) | 0.103 | -0.624(-1.042,-0.206) | 0.004 |  |
| Black | -0.037(-0.158,0.084) | 0.541 |  | ref | 0.059(-0.749,0.867) | 0.884 | -0.231(-1.062,0.601) | 0.577 | -0.118(-0.723,0.487) | 0.694 |  |
| Mexican American | -0.054(-0.247,0.139) | 0.563 |  | ref | -1.265(-2.873,0.343) | 0.114 | 0.259(-1.125,1.643) | 0.695 | -0.843(-1.893,0.208) | 0.108 |  |
| Others | 0.052(-0.159,0.263) | 0.62 |  | ref | 0.265(-0.640,1.171) | 0.553 | -0.035(-0.912,0.841) | 0.935 | -0.086(-1.156,0.983) | 0.87 |  |
| Marriage |  |  |  |  |  |  |  |  |  |  | 0.537 |
| Married/Living with partner | -0.056(-0.168,0.056) | 0.324 | 0.76 | ref | -0.464(-0.872,-0.055) | 0.027 | -0.315(-0.736, 0.105) | 0.14 | -0.298(-0.762, 0.165) | 0.204 |  |
| Widowed/divorced/separated | -0.1(-0.240,0.040) | 0.157 |  | ref | -0.458(-1.051, 0.135) | 0.128 | -0.46(-1.039, 0.118) | 0.117 | -0.874(-1.533,-0.214) | 0.01 |  |
| Never married | -0.028(-0.156,0.099) | 0.655 |  | ref | -0.08(-0.747,0.587) | 0.807 | 0.053(-0.616,0.722) | 0.873 | -0.452(-1.205,0.302) | 0.231 |  |
| Poverty |  |  |  |  |  |  |  |  |  |  | 0.637 |
| High(＞3.5) | -0.043(-0.203,0.117) | 0.59 | 0.068 | ref | -0.416(-1.087,0.256) | 0.219 | -0.269(-0.913,0.375) | 0.405 | -0.1(-0.935,0.734) | 0.81 |  |
| Middle(＞1.3and≤3.5) | 0.02(-0.107,0.148) | 0.75 |  | ref | -0.223(-0.712,0.265) | 0.366 | 0.021(-0.518,0.561) | 0.937 | -0.342(-0.930,0.246) | 0.25 |  |
| Low(≤1.3) | -0.198(-0.307,-0.088) | <0.001 |  | ref | -0.561(-1.113,-0.009) | 0.046 | -0.538(-1.043,-0.033) | 0.037 | -0.86(-1.364,-0.357) | 0.001 |  |
| Education |  |  |  |  |  |  |  |  |  |  | 0.477 |
| >High school | -0.071(-0.176,0.034) | 0.185 | 0.981 | ref | -0.477(-0.901,-0.053) | 0.028 | -0.337(-0.807, 0.133) | 0.157 | -0.381(-0.906, 0.144) | 0.153 |  |
| High school | -0.068(-0.257,0.122) | 0.478 |  | ref | -0.099(-0.785, 0.586) | 0.773 | -0.361(-0.976, 0.254) | 0.245 | -0.721(-1.413,-0.028) | 0.042 |  |
| < High school | -0.089(-0.235,0.057) | 0.227 |  | ref | -0.583(-1.126,-0.040) | 0.036 | -0.017(-0.639, 0.606) | 0.957 | -0.615(-1.265, 0.035) | 0.063 |  |
| Diabetes |  |  |  |  |  |  |  |  |  |  | 0.99 |
| Yes | -0.04(-0.400,0.320) | 0.824 | 0.819 | ref | -0.431(-1.733,0.871) | 0.505 | -0.158(-1.376,1.059) | 0.793 | -0.548(-2.001,0.905) | 0.448 |  |
| No | -0.08(-0.156,-0.004) | 0.039 |  | ref | -0.394(-0.714,-0.074) | 0.016 | -0.3(-0.653, 0.052) | 0.094 | -0.522(-0.864,-0.180) | 0.003 |  |
| Hypertension |  |  |  |  |  |  |  |  |  |  | 0.054 |
| Yes | -0.099(-0.200,0.001) | 0.053 | 0.518 | ref | -0.846(-1.259,-0.433) | <0.001 | -0.413(-0.848, 0.021) | 0.062 | -0.658(-1.155,-0.161) | 0.01 |  |
| No | -0.052(-0.159,0.056) | 0.342 |  | ref | -0.087(-0.422,0.248) | 0.606 | -0.274(-0.672,0.123) | 0.173 | -0.414(-0.852,0.023) | 0.063 |  |
| CVD |  |  |  |  |  |  |  |  |  |  | 0.236 |
| Yes | -0.229(-0.337,-0.121) | <0.0001 | 0.008 | ref | -1.014(-1.736,-0.292) | 0.007 | -0.83(-1.387,-0.272) | 0.004 | -1.127(-1.759,-0.495) | <0.001 |  |
| No | -0.048(-0.132,0.037) | 0.266 |  | ref | -0.309(-0.638, 0.020) | 0.065 | -0.202(-0.552, 0.149) | 0.257 | -0.424(-0.817,-0.031) | 0.035 |  |
